# Adjacent Fu’s subcutaneous needling as an adjunctive healing strategy for diabetic foot ulcers: Two case reports

**DOI:** 10.1097/MD.0000000000032271

**Published:** 2022-12-16

**Authors:** Fei Qi, Huiyi Huang, Yanyan Cai, Zhonghua Fu

**Affiliations:** a Clinical Medical College of Acupuncture & Moxibustion and Rehabilitation, Guangzhou University of Chinese Medicine, Guangzhou, Guangdong, China; b Foshan hospital of Chinese Medicine, Foshan, Guangdong, China; c The Institute of Fu’s Subcutaneous Needling, Beijing University of Chinese Medicine, Beijing, China.

**Keywords:** adjunctive healing strategy, case report, diabetic foot ulcers, Fu's subcutaneous needling

## Abstract

Diabetic foot ulcers (DFUs) present with different grades of ischemia and infection and are associated with high mortality and disability rates with little effective treatment. We used Fu Subcutaneous Needling (FSN) to treat 2 cases with DFUs and achieved satisfactory results.

**Patient concerns:**

Two cases of DFUs showed poor recovery after conventional wound care treatment, and case 2 was confronted with the risk of amputation.

**Diagnosis:**

Two patients with history of diabetes were diagnosed with DFUs, presenting with lower leg and foot ulcers.

**Interventions:**

Case 1 received 6 sessions of FSN treatment in 8 days, and case 2 received 10 sessions of FSN treatment in 14 days.

**Outcomes:**

Case 1 completely healed from a 1 × 0.5-cm blister and a 0.5 × 0.5-cm ulcer of the right lower leg 14 days after the first FSN treatment. The ulcer area of the left foot in case 2 decreased from 6 × 7 cm to 4 × 3.5 × 0.2 cm. Three months of follow-up revealed full wound closure.

**Lessons:**

FSN is effective for healing with DFUs, and it may be used as an adjunctive healing strategy for DFUs patients when conventional treatments such as infection, glycemic control, and local ulcer care are not available.

## 1. Introduction

Diabetic foot ulcers (DFUs) are common and one of the most serious complications of diabetes mellitus, with varying grades of ischemia and infection.^[1]^ DFUs are among the main causes of disability and death in diabetic patients. With the incidence of diabetes gradually increasing worldwide, the World Health Organization reported that approximately 422 million people have diabetes, 15 to 25% of whom will undergo DFUs.^[2–4]^ According to statistics, 40 to 60% of non traumatic amputations of the lower limbs are performed in diabetic patients worldwide.^[5]^ The high incidence of disability and morbidity due to DFUs has become an important public health problem. Glycemic control, wound care, and prompt revascularization are general treatments, although with poor healing rates.^[6]^ Adjunctive therapies for DFUs such as acupuncture, massage, and acupoint injection have shown positive effects.^[7]^

**Figure 1.**
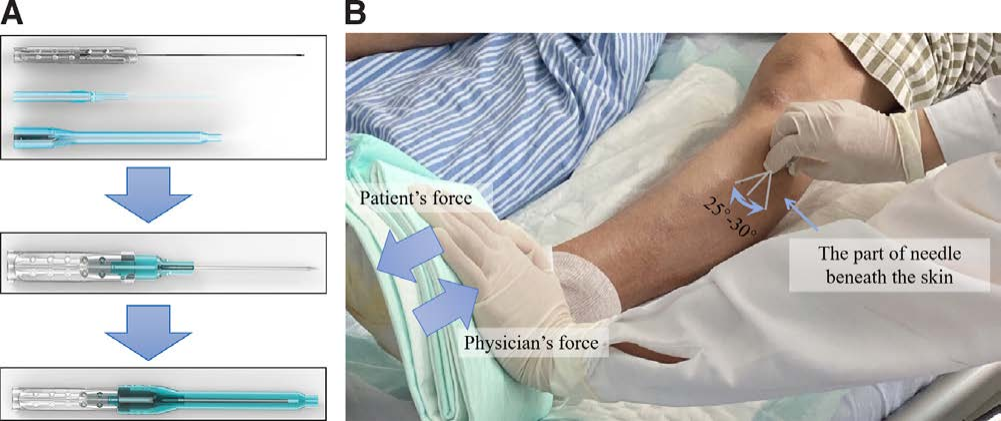
(A) The structure of FSN needle designed and patented in China (patent number: CN97114318, Nanjing FSN Medical Appliances Co, China). (B) The insertion point is at around 10 cm below the left knee in case 1, where swaying movement is performed beneath the skin, and combined with the reperfusion approach which physician resisted patient’s movement of ankle flexion. FSN = Fu's subcutaneous needling.

Fu's subcutaneous needling (FSN) has been an innovative acupuncture therapy since 1996 (Fig. 1A).^[8]^ FSN is used to stimulate the subcutaneous layer, which is connected to the muscle layer to improve blood circulation, and is applied for painful musculoskeletal problems with positive effects.^[9,10]^ Herein we report 2 cases of DFUs that were effectively treated with FSN.

## 2. Case presentation

### 2.1. Case 1

**Figure 2.**
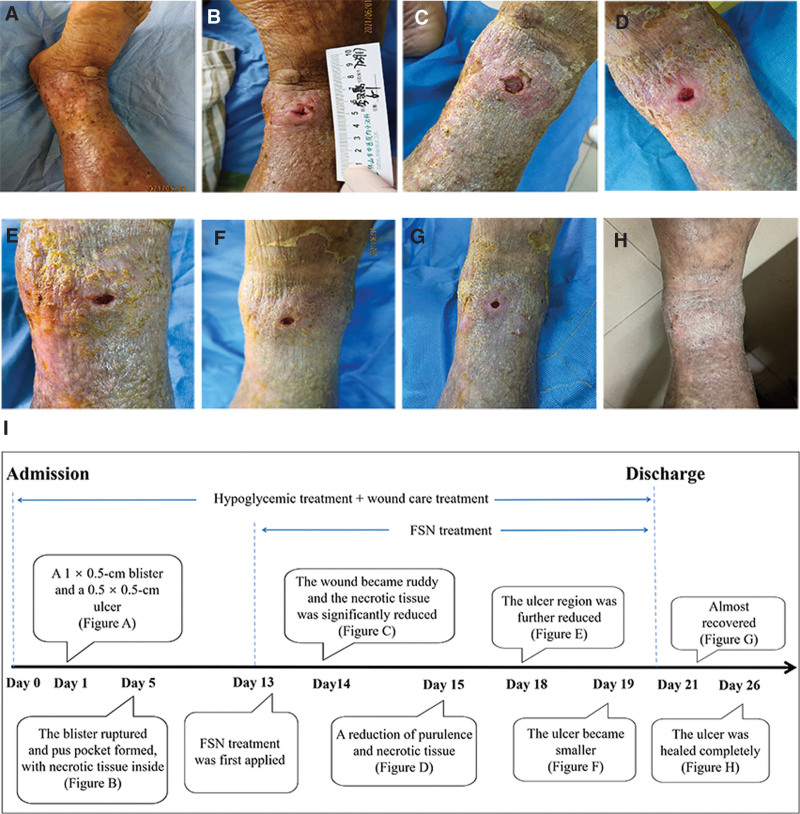
Wound conditions of case 1 in the (A) 1st, (B) 5th, (C) 14th, (D) 15th, (E) 18th, (F) 19th, (G) 21st, and (H) 26th day. (I) The timeline of healing process with FSN treatment from admission to completely healed. FSN = Fu's subcutaneous needling.

A 71-year-old man with a history of diabetes for over 6 years was admitted to the Foshan Hospital of Traditional Chinese Medicine for aggravating swelling, pain, and increased exudate of the right ankle dorsum on May 28, 2021. The patient had a 22-year-long history of hypertension, which was well controlled by losartan and nifedipine tablets. Although repaglinide (2 mg oral administration, 3 times a day) and metformin (0.5 g oral administration, 3 times a day) were administered before admission, the patient had poor glycemic control. The patient presented with swelling, decreased skin pliability, and coldness of his right lower leg for 2 years, which aggravated a week prior to hospitalization. His right foot became swollen with pain and a blister grew on the surface of the skin (Fig. 2A). Physical examination showed a 1 × 0.5-cm blister and a 0.5 × 0.5-cm ulcer on his right ankle, without odor or pruritus. The general test showed normal body temperature (36.8ºC) and blood pressure (130/70 mm Hg). Neurological examination revealed decreased pain and temperature in the right lower limb. Arteriovenous color ultrasound examination revealed swollen subcutaneous tissue and poor blood circulation in his right leg. Routine blood tests (May 29, 2021) revealed a white blood cell count of 6.00 × 10^9^/L; red blood cell count of 3.93 × 10^12^/L, hemoglobin of 121 g/L, and hematocrit of 37.4%. Hemoglobin A1c level was 7.3%. Examination of pus and wound secretions revealed no bacterial infection. Based on this evidence, the patient was initially diagnosed with an uninfected diabetic foot ulcer (Grade I of the Wagner classification; Grade I of the international working group on the diabetic foot classification).^[11]^

After admission, the patient received conventional treatments, such as hypoglycemic treatment, including subcutaneous injection of insulin aspart (6 IU, 3 times a day), rapid-acting insulin (during each meal), and metformin hydrochloride tablets (500 mg oral administration, 3 times a day) to control glucose. Moreover, irrigation with 3% boric acid solution, debridement, and dressing treatment were applied daily for wound care. On the 5th day of admission, the blister ruptured and a pus cavity formed, with necrotic tissue inside (Fig. 2B). However, necrotic tissue reappeared over the subsequent few days (Fig. 2C). Therefore, the patient began to receive FSN as an adjunctive treatment on the 13th day of admission, with a frequency interval of 1 or 2 days. The detailed steps of the FSN treatment are as follows.^[8]^ First, the myofascial trigger points (MTrPs) within the muscle belly were palpated and felt tight and less elastic by the practitioner’s fingers.^[8]^ In these 2 cases, MTrPs were found in the ipsilateral tibialis anterior and gastrocnemius muscles. The FSN needle was then inserted at an angle of 15 to 25° from the skin into the subcutaneous layer around the belly of the muscles. The swaying movement was performed at a frequency of approximately 100 times per minute for 2 minutes. Meanwhile, the reperfusion approach was performed with muscle movement to cause contraction against the physician’s resistance for approximately 8 seconds. For example, ankle flexion is a type of contraction of the tibialis anterior (Fig. 1B), and ankle extension is the contraction of the gastrocnemius.

The tissue around the ulcers regained a ruddy appearance after the first FSN treatment (Fig. 2D), the necrotic tissue was quickly reduced in the following few days (Fig. 2E–G), and finally disappeared completely after 6 sessions of FSN treatment within 8 days (Fig. 2H). A timeline of the healing process is shown in Fig. 2I.

### 2.2. Case 2

**Figure 3.**
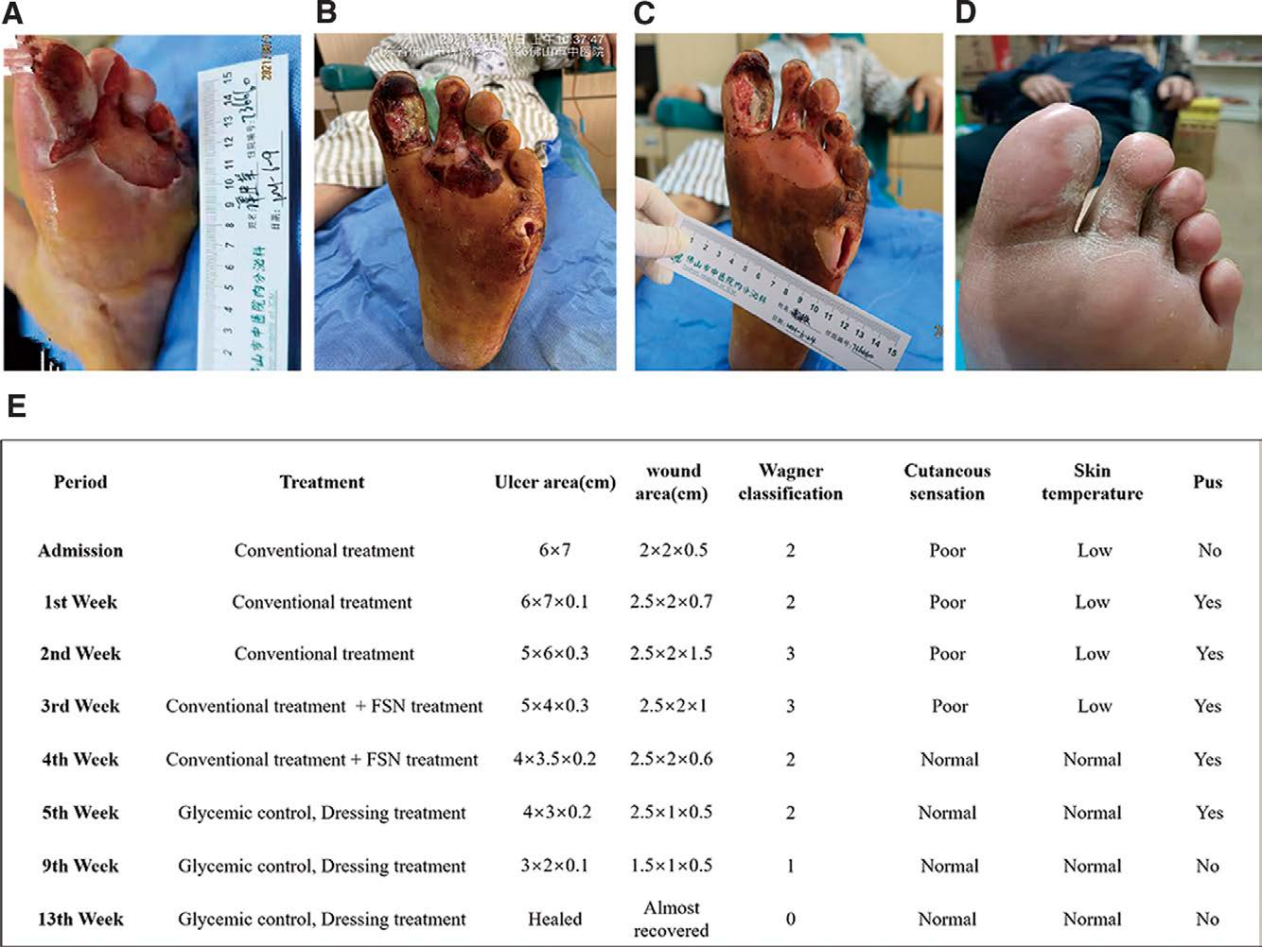
(A) The day of admission, an ulcer measuring 6 × 7 cm on the plantar aspect of the left forefoot, and an abscess measuring 2 × 2 × 0.5 cm on the lateral aspect of the left foot. (B) The first day of FSN treatment, the ulcers with necrotic tissues eats into tendons, muscle or bone on the plantar of the first to third toes, and the deep abscess was measuring 2 × 2 × 0.7 cm. (C) Six days after continuous FSN treatment, the wound appeared ruddy and dry with reduced necrotic tissues and exudation. (D) Three months after FSN treatment, the wound demonstrated full closure. (E) Wound baseline characteristics and healing process of the patient (the conventional treatments including infection and glycemic control and local ulcer care.). FSN = Fu's subcutaneous needling.

A 48-year-old man was admitted to the same hospital as case 1 with the complaining of 2 deteriorative purulent ulcers on his left foot for 2 months. He also had parethesia, such as coldness and numbness in both lower legs. The patient had a 7-year-long history of diabetes. Physical examination revealed a 6 × 7-cm blister on the plantar aspect of the forefoot and a 2 × 2 × 0.5-cm abscess on the lateral aspect (Fig. 3A). Physical examination revealed decreased pain and thermal sensation. Related laboratory tests showed the following results: Hemoglobin A1c: 12.8%; white blood cell count: 10.75 × 10^9^/L; neutrophils: 7.93 × 10^9^/L; red blood cell count: 5.81 × 10^12^/L; albumin-creatinine ratio: 2.8 mg/mmol; microalbumin: 29.2 mg/L; glucose: 7.89 mmol/L; fructosamine: 2.18 L. Urine rountine test: glucose: 3+; uric bravery former: 1+; leukocyte esterase 2+. A highly resistant strain of Methicillin-resistant *Staphylococcus aureus* was detected by bacterial culture of pus secretions. Magnetic resonance imaging revealed swelling of the surrounding subcutaneous soft tissue. A diagnosis of diabetic foot with mild infection was made (Grade II of Wagner classification; Grade II of international working group on the diabetic foot classification).^[11]^

Drug sensitivity test showed that levofloxacin was the most effective antibiotic against Methicillin-resistant *S. aureus* (June 9, 2021). The patient was administered levofloxacin (0.25 g oral, twice a day) for 14 days. *Providencia stuartii*, *Pseudomonas Aeruginosa* and *Myroides* were detected in pus and wound secretion examination after 14 days, and piperacillin-tazobactam (4.5 g intravenous injection, twice a day) was used as the most sensitive antibiotic for another 14 days. Besides, continuous insulin aspart was used in insulin pump (1.4 U/h in 7 am–7 pm and 0.7 U/h in 7 pm–7 am) to control glucose, and 3% boric acid solution irrigation and dressing treatment were used to treat wound infection locally every day. Based on the condition that bone and tendon exposure appeared from the bottom of the left big toe after debridement (Fig. 3B), amputation of the left big toe was suggested by consultation if the condition continued to worsen. Despite good glucose control and daily wound management, pus still flowed from the abscess.

In search of a better treatment, FSN treatment was applied once a day from the 11th day of admission. The detailed steps were performed in a manner similar to case 1. The excretion decreased, and the necrotic tissue of the ulcers decreased from 25 to 20% (Fig. 3C). In the 4th week of admission, FSN intervention was performed once every 2 days. The ulcer decreased to 4 × 3.5 × 0.2 cm, and appeared more ruddy and dry with little necrotic tissue existed. Thus, the patient was discharged on the 5th week and regularly cleaned and dressed every 1 to 3 days until the ulcer healed entirely. At the 3-month follow-up, the wound demonstrated full closure with smooth scar tissue (Fig. 3D). The patient was grateful that the FSN treatment saved him from amputation. The baseline characteristics and healing process of the patient are shown in Figure 3E.

## 3. Discussion

Diabetic foot lesions develop following injury and are usually accompanied by peripheral neuropathy and arterial diseases.^[12]^ Impaired microvascular reactivity diminishes the blood supply to ulcerated areas,^[13]^ and insufficient vascularization, tissue hypoxia, and a high probability of infection contribute to delayed wound healing.^[7]^ For these 2 cases, case 2, especially poor glycemic control and inadequate microcirculation, led to severe DFUs. However, conventional treatments such as infection, glycemic control, and local ulcer care failed to promote ulcers healing in these 2 cases, suggesting that a sufficient supply of blood and oxygen is essential for accelerating tissue healing.^[14]^

Ischemia is the underlying cause of chronic muscle spasm.^[15]^ MTrPs are hard, palpable nodules in a taut band of skeletal muscle, which were palpated in the tibialis anterior and gastrocnemius in the 2 cases, revealing that muscles related to ischemic areas appear to be in tightened condition. FSN is a therapy that inactivates MTrPs to relieve muscle spasticity and dilate the blood vessels, which are compressed by tightened muscles to promote local blood circulation.^[16]^ Moreover, with muscle movement of contraction of relaxation in FSN treatment, the combination of vasodilation and increase in tissue blood flow may potentially accelerate the production of collagen fibers and granulation tissue and facilitate ulcer healing.^[17]^

This case report provides promising evidence that FSN is an adjunctive strategy to treat DFUs because of its unique features: needling points are remote from the ulcerative area and are therefore much less likely to increase the risk of infection; although the FSN needle does not reach the muscle layer, it can act on muscles by performing only in the subcutaneous layer, which surrounds and separates the muscles.^[18]^

### 3.1. Limitations

These were 2 successful cases of accelerated DFUs healing caused by FSN. Further randomized controlled trials are needed to prove evidence for FSN to be an adjunctive therapy and to help millions of people suffering from DFUs.

## 4. Conclusion

In addition to conventional treatments, including infection, glycemic control, and local ulcer care, FSN can effectively inactivate MTrPs to relieve tightened muscles and promote local blood circulation, which may accelerate the production of collagen fibers and granulation tissue, facilitate ulcer healing, and reduce the risk of amputation.

## Acknowledgments

We are grateful to the patients for their participation in the study and allowing us to publish these case reports. We thank Dr Alexander B. Mearns, Private Clinic, Tain, Ross-Shire, Scotland for his excellent technical assistance.

## Author contributions

**Conceptualization:** Fei Qi, Huiyi Huang.

**Investigation:** Fei Qi.

**Methodology:** Fei Qi, Huiyi Huang, Yanyan Cai.

**Project administration:** Fei Qi, Huiyi Huang, Zhonghua Fu.

**Supervision:** Huiyi Huang, Zhonghua Fu.

**Validation:** Fei Qi.

**Visualization:** Fei Qi, Huiyi Huang

**Writing – original draft:** Fei Qi, Yanyan Cai.

**Writing – review & editing:** Huiyi Huang, Zhonghua Fu.
